# Efficacy of low-dose cone beam computed tomography and metal artifact reduction tool for assessment of peri-implant bone defects: an in vitro study

**DOI:** 10.1186/s12903-022-02663-8

**Published:** 2022-12-17

**Authors:** Alaa Shafiek Nomier, Yousria Salah El-Din Gaweesh, Maha R. Taalab, Shaimaa Abu El Sadat

**Affiliations:** 1 Department of Oral Medicine, Periodontology, Oral Diagnosis and Oral Radiology, Faculty of Dentistry, Alexandria University, Champolion St. Azarita, Alexandria, 21521 Egypt; 2 Department of Oral and Maxillofacial Radiology, Faculty of Dentistry, Ain-Shams University, Cairo, Egypt

**Keywords:** CBCT, Dehiscence, Dental implants, Fenestration, Low-dose protocol, MAR

## Abstract

**Background:**

Early accurate radiographic assessment of peri-implant bone condition is highly important to avoid excessive loss of supporting bone and implant failure. Cone beam computed tomography (CBCT) is the radiographic technique of choice if peri-implant dehiscence and fenestration defects are suspected. The higher radiation dose and the presence of beam hardening artifacts are the main drawbacks of CBCT imaging techniques. This study aims to evaluate the influence of low-dose cone beam computed tomography (LD-CBCT) and metal artifact reduction (MAR) tool on the assessment of peri-implant dehiscence and fenestration.

**Methodology:**

Thirty titanium implants were inserted into bovine rib blocks. Twenty had standardized bone defects (10 with dehiscence and 10 with fenestration), while the remaining 10 were used as control group with no defects. Radiographic examinations held with high‐definition CBCT (HD-CBCT) and LD-CBCT with and without application of MAR tool. Images were assessed by four examiners for the presence or absence of peri-implant defects. The area under the area under the receiver operating characteristic (ROC) curve (AUC), sensitivity, specificity, and accuracy were calculated for all radiographic protocols.

**Results:**

In the absence of MAR tool, there was no difference in AUC and diagnostic values between LD-CBCT and HD-CBCT for detection of both defects. When the MAR tool was applied, the AUC values, sensitivity, and accuracy were higher in HD-CBCT than in LD-CBCT for the detection of both defects, especially for the dehiscence, while specificity remained the same.

**Conclusion:**

LD-CBCT can be used in the evaluation of peri-implant dehiscence and fenestration without any decrease in diagnostic accuracy. The application of MAR tool decrease the diagnostic ability of both defects, especially for the detection of dehiscence defects.

## Background

Since the introduction of the Bränemark implant system, dental implants have become an increasingly popular therapeutic modality for tooth loss [1]. Unfortunately, the success rate of functional implants and their prosthetic restorations is influenced by biological and mechanical conditions. The success of implant therapy is dependent on the absence of mobility, soft tissue inflammation, abscess, or pain [2]. Appropriate bone volume around the implants is essential for the primary stability of the implant. So, it has been reported that it is critical to have at least 1 mm of bone surrounding the implant in all directions. Inability to obtain complete coverage of the implant by bone may increase the risk of peri-implant mucositis, periimplantitis, and peri-implant defects [2, 3].

Insufficient amounts of bone surrounding the implants and incorrect position of the dental implant significantly increase the probability of peri-implant bone defects such as dehiscence and fenestration. Dehiscence and fenestration defects involve bone denudation over the cervical or radicular implant surface. The absence of bone from the implant’s cervical portion is called peri-implant dehiscence, while peri-implant fenestration is the absence of bone in a portion of the implant with the implant’s coronal third is covered by bone [4, 5].

It was thought that the presence of peri-implant defects like dehiscence and fenestration would affect the survival and success rate of the dental implant and could cause total loss of the implant. The extent of peri-implant bone loss and the configuration of the defect have a major impact on the treatment outcomes of peri-implantitis [5, 6]. As a result, early accurate radiographic assessment of bone defects surrounding dental implants is crucial [7].

Radiographic imaging plays an important role in the evaluation of peri-implantitis and peri-implant bone defects. Intraoral periapical radiography with the parallel technique is the gold standard radiographic technique for postsurgical assessment of dental implants. It has a low patient radiation dose, low cost, and high resolution [8]. However, it is not appropriate for detecting crestal bone loss in the buccal and lingual aspects of dental implants because of the two-dimensional (2D) representation of the three-dimensional (3D) anatomical structures. This modality is only appropriate for assessing the interproximal bone level and it is not useful in determining whether peri-implant dehiscence or fenestration is present or not [7, 9, 10].

Cone beam computed tomography (CBCT) was introduced into the dento-maxillofacial field to overcome the limitations of two-dimensional imaging techniques [11]. It enables the visualization of buccal and lingual cortical bone around implants. Using CBCT improves the diagnostic accuracy at the expense of greater cost and radiation exposure. The higher diagnostic accuracy allows earlier detection of bone defects and interventions to control further bone loss [7, 12]. According to the ALADA principle (As Low As Diagnostically Acceptable), CBCT must be justified based on an individual's needs and provide benefits to the patient that outweigh the risks [13, 14].

Low-dose cone beam computed tomography (LD-CBCT) protocols have recently become available, which can reduce exposure factors without sacrificing image quality for diagnostic purposes. It can be done by either using pre-set dose reduction settings or manually adjusting scanning parameters provided by the machine. Low-dose techniques can be accomplished by decreasing the tube voltage, tube current, scanning time, projection image number, using an incomplete rotation angle (180° rather than 360° rotation), and/or increasing the voxel size. For optimization of radiation dose, researchers preferred tube current reduction over kVp reduction for achieving acceptable image quality with CBCT [15–17].

In addition to the higher radiation dose and cost, image artifacts is considered one of the drawbacks of CBCT imaging technique. Beam hardening, noise, and scattered radiation can decrease the quality of CBCT images. Metal artifacts produced by high density objects like dental titanium implants, cause beam hardening and streaking artifacts. The selective attenuation of low-energy x-ray photons by allowing the passage of high-energy photons is called beam hardening artifact, which results in a dark band surrounding hyperdense object. Streaking artifact is caused by scattered radiation from hyperdense objects which is seen as hyperdensity lines arising from the metallic object [18, 19]. These artifacts affect the visibility of areas surrounding dental implants, therefore, may affects the diagnostic ability of CBCT in the detection of peri-implant bone defects [20].

According to the literature, different methods have been used to decrease metal artifacts in CBCT images, such as using metallic filters, anti-scatter grids, or choosing a smaller field of view. Moreover, newer metal artifact reduction (MAR) algorithms have been introduced to minimize the artifacts of beam hardening in the final images and to improve image quality [21]. The use of MAR algorithms for artifact reduction is becoming increasingly common. The effect of MAR algorithms on different diagnostic tasks are now being investigated by many researchers, and the findings are still controversial.

There are not enough studies that clarify the effect of using the LD-CBCT protocol and MAR algorithm in the assessment of peri-implant bone defects [12, 15]. Thus, the primary aim of this study was to compare the diagnostic accuracy of low-dose cone beam computed tomography (LD-CBCT) to high-definition cone beam computed tomography (HD-CBCT) in the evaluation of peri-implant fenestration and dehiscence defects. The secondary aim was to investigate the effect of using the metal artifact reduction (MAR) algorithm in addition to both the LD-CBCT and HD-CBCT on the detection of peri-implant bone defects.

## Methods

### Sample selection and preparation

This in vitro diagnostic accuracy study was planned and performed according to STARD guidelines [22]. Fresh bovine ribs were acquired from a local butcher and prepared by removing the overlying soft tissue, then divided into 30 blocks of equal dimensions (20 mm in length). The width of the superior surface of each block was measured, and only blocks with a width of 7 to 10 mm were chosen [23]. Dried ribs, ribs with pre-existing defects, and very thick or thin ribs were excluded [2, 7, 9]. The sample size was calculated to be 30 (10 for the control group, 10 for the fenestration group, and 10 for the dehiscence group) with an alpha error of 5% and a study power of 80% [7, 24].

The blocks were flattened by performing an osteotomy on the superior and inferior borders of the block. The buccal plate of bone was represented by the anterior or convex surface of the rib, whereas the lingual plate of bone was represented by the posterior or concave surface. The bovine blocks were kept frozen during all the study procedures to preserve the bone marrow's hydration and integrity [7, 9].

**Fig. 1:**
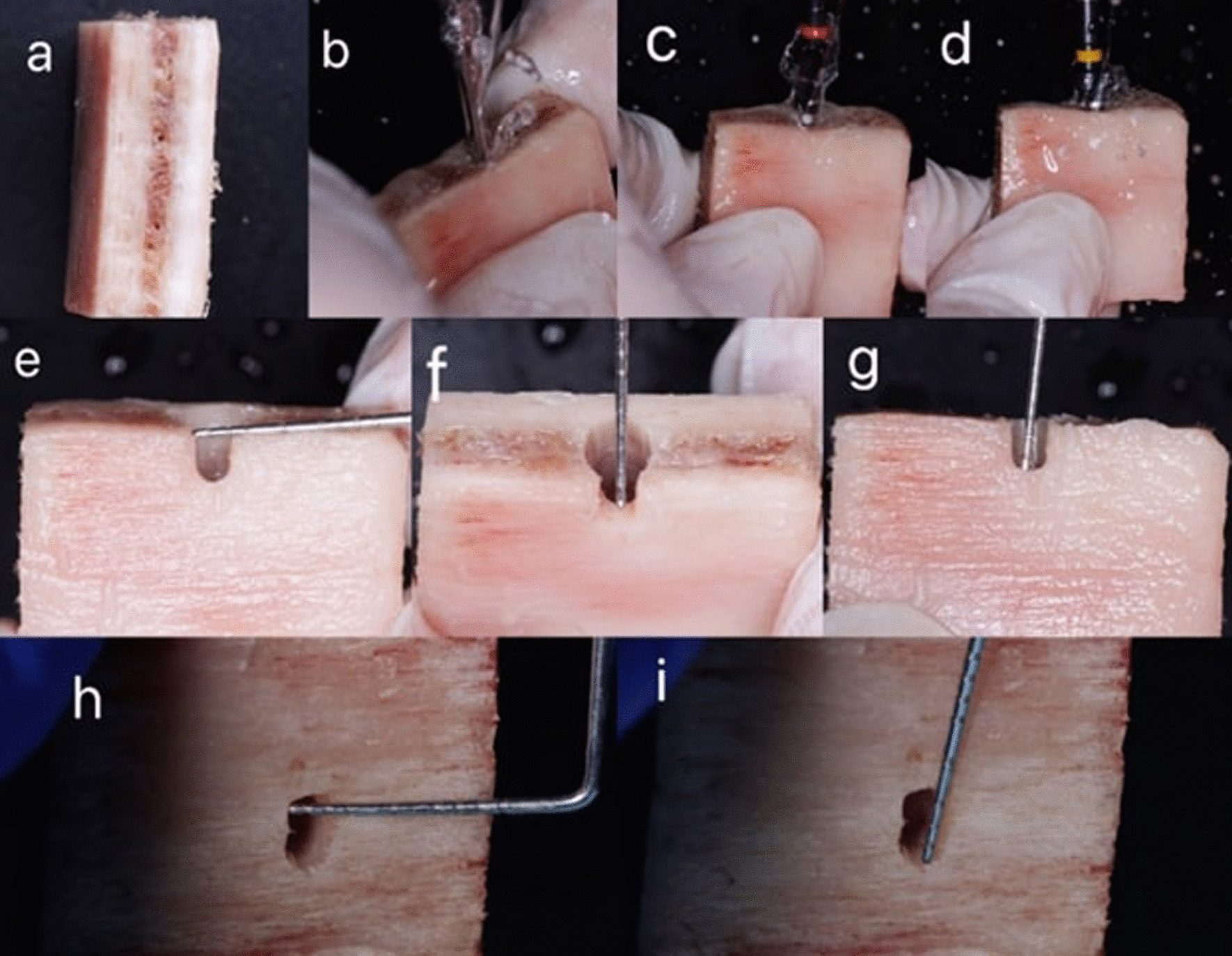
Sample preparation and defect creation. **a** bovine rib block before implant osteotomy. **b**–**d** implant site preparation with drilling sequence. **e**–**g** dehiscence defect creation. **h**, **i** fenestration defect creation

Implant osteotomies measuring 3.6 mm × 12 mm were prepared in the blocks following the manufacturer's drilling sequence. After implant site preparation, the bone blocks were randomly divided into three equal groups: the dehiscence defect group, the fenestration defect group, and the no defect control group. The blocks of the three groups were assigned different numbers randomly. Then the defects were created mechanically by a round diamond bur (MANI, Tochigi, Japan). Dehiscence defects were prepared as half-elliptical defects on the superior edge of the buccal sides where the cervical portion of the implant would be inserted. The fenestration defects were prepared on the buccal sides of the bone blocks 10 mm from the superior edge as an elliptical defect form. Each defect was prepared with care not to exceed 2 mm in width and 3 mm in length, as this is the important threshold used by previous research **(**Fig. 1) [2, 23, 25].

**Fig. 2:**
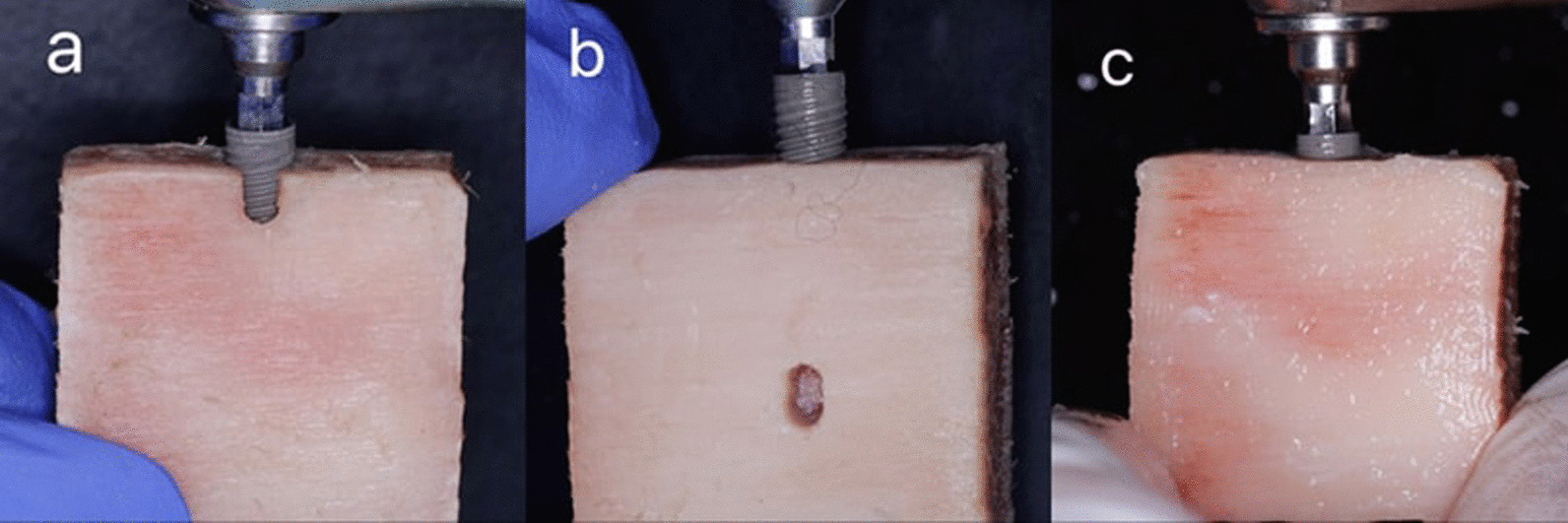
Insertion of titanium implants into the three groups of the blocks (dehiscence, fenestration, control)

Following the creation of bone defects, 30 titanium implants (Dual Implant, Titan Industries, Egypt®) measuring 3.6 mm × 12 mm were inserted into the osteotomies of all the bovine rib blocks. In the control group, implants were inserted in the absence of any defect. Just one implant was placed into each bovine rib block to prevent image quality degradation due to metal artifacts from nearby implants. After that, the bone blocks were coated with a 1.5 mm layer of pink wax to mimic the soft tissue surrounding the alveolar bone and to maintain an equivalent x-ray beam attenuation (Fig. 2) [3, 26, 27].

### Radiographic examination

To standardized the position of bone blocks within the field of view during the radiographic examination procedure, a piece of plastic foam with a central groove was developed to accommodate all the bone blocks [9].

**Fig. 3:**
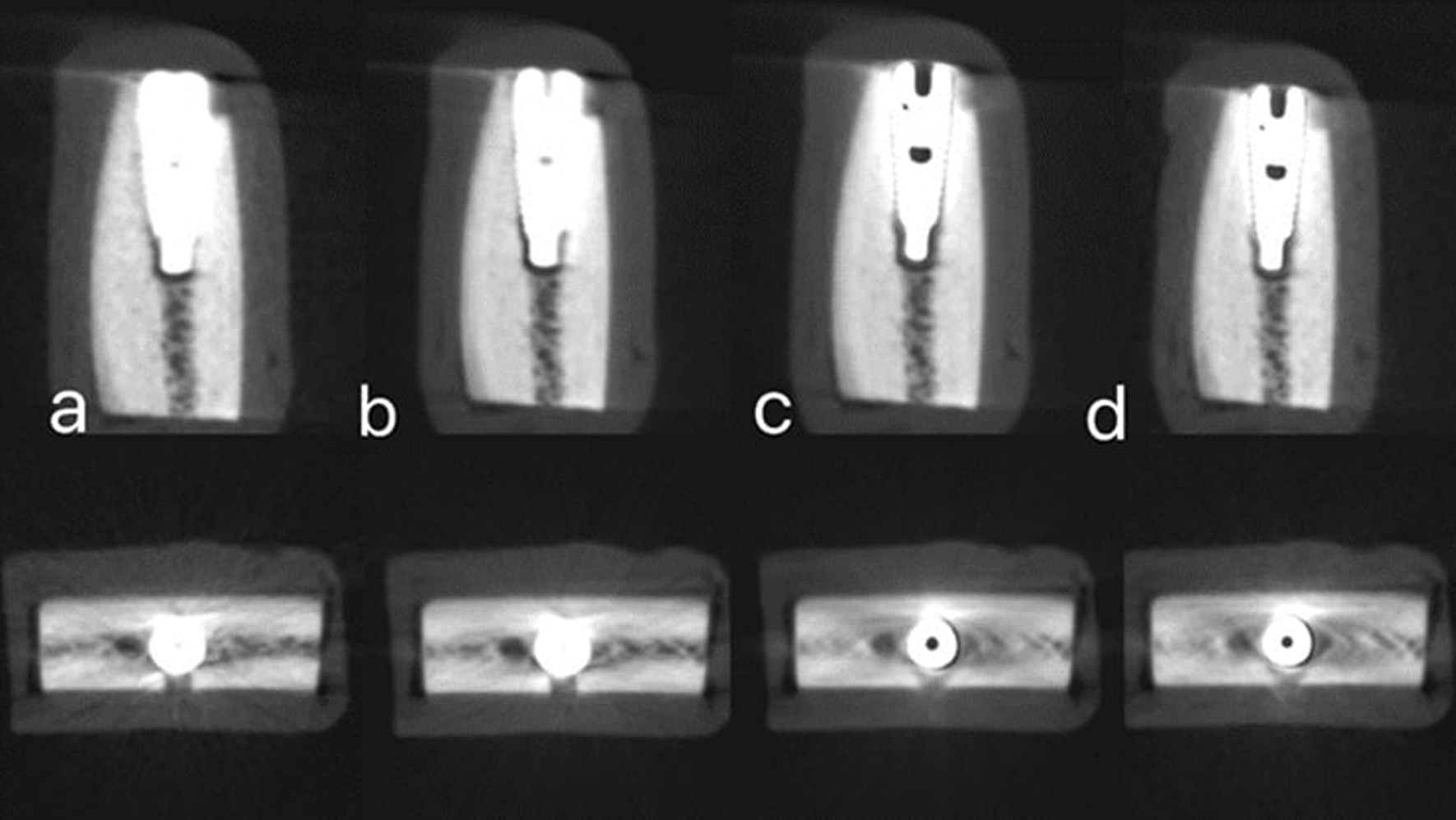
Sagittal and axial images with simulated peri-implant dehiscence defect obtanied with different four radiographic protocols. **a** High-dose CBCT without MAR. **b** Low-dose CBCT without MAR. **c** High-dose with MAR. **d** Low-dose CBCT with MAR

**Fig. 4:**
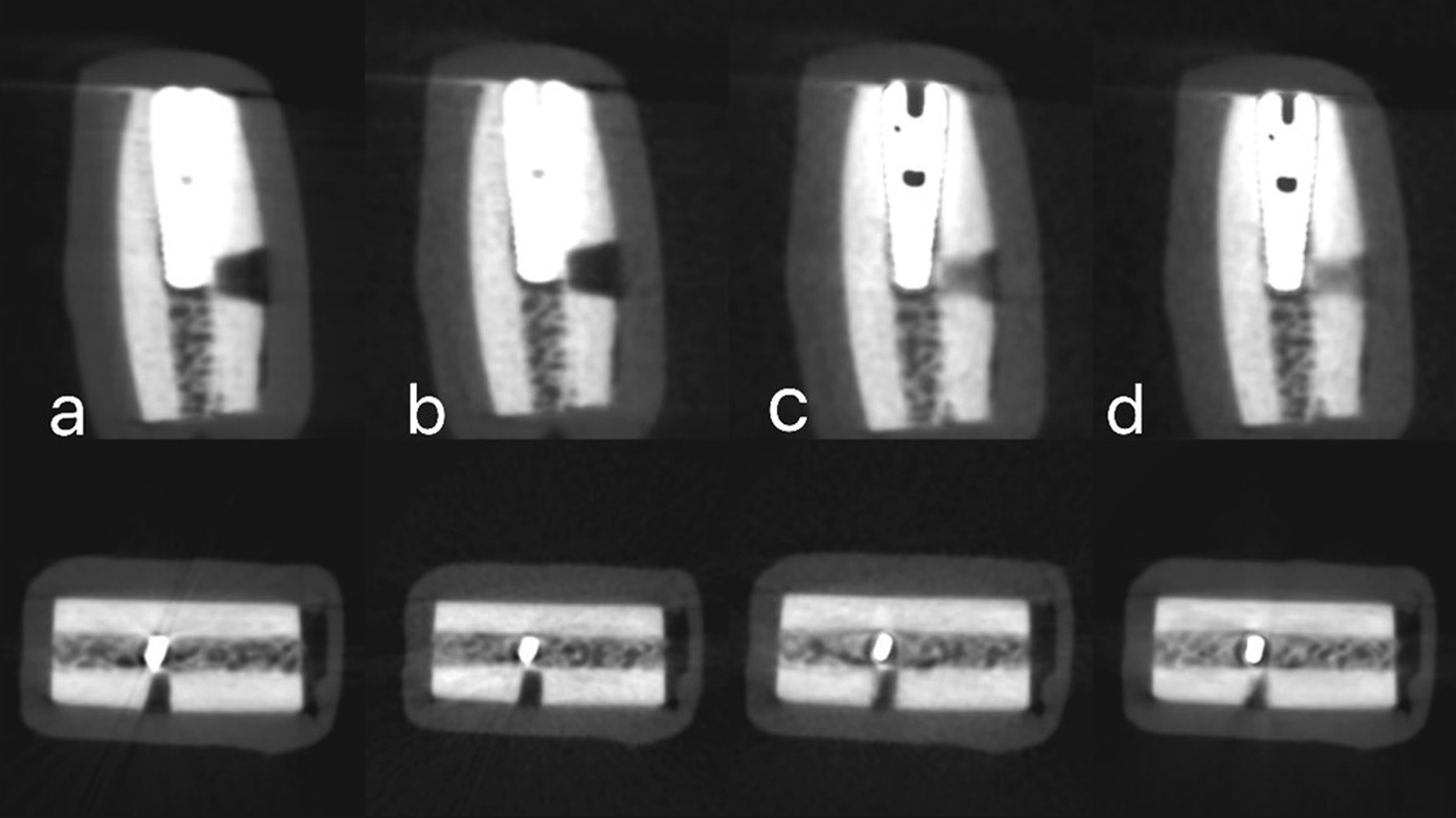
Sagittal and axial images with simulated peri-implant fenestration defect obtained with different four radiographic protocols. **a** High-dose CBCT without MAR. **b** Low-dose CBCT without MAR. **c** High-dose with MAR. **d** Low-dose CBCT with MAR

**Fig. 5:**
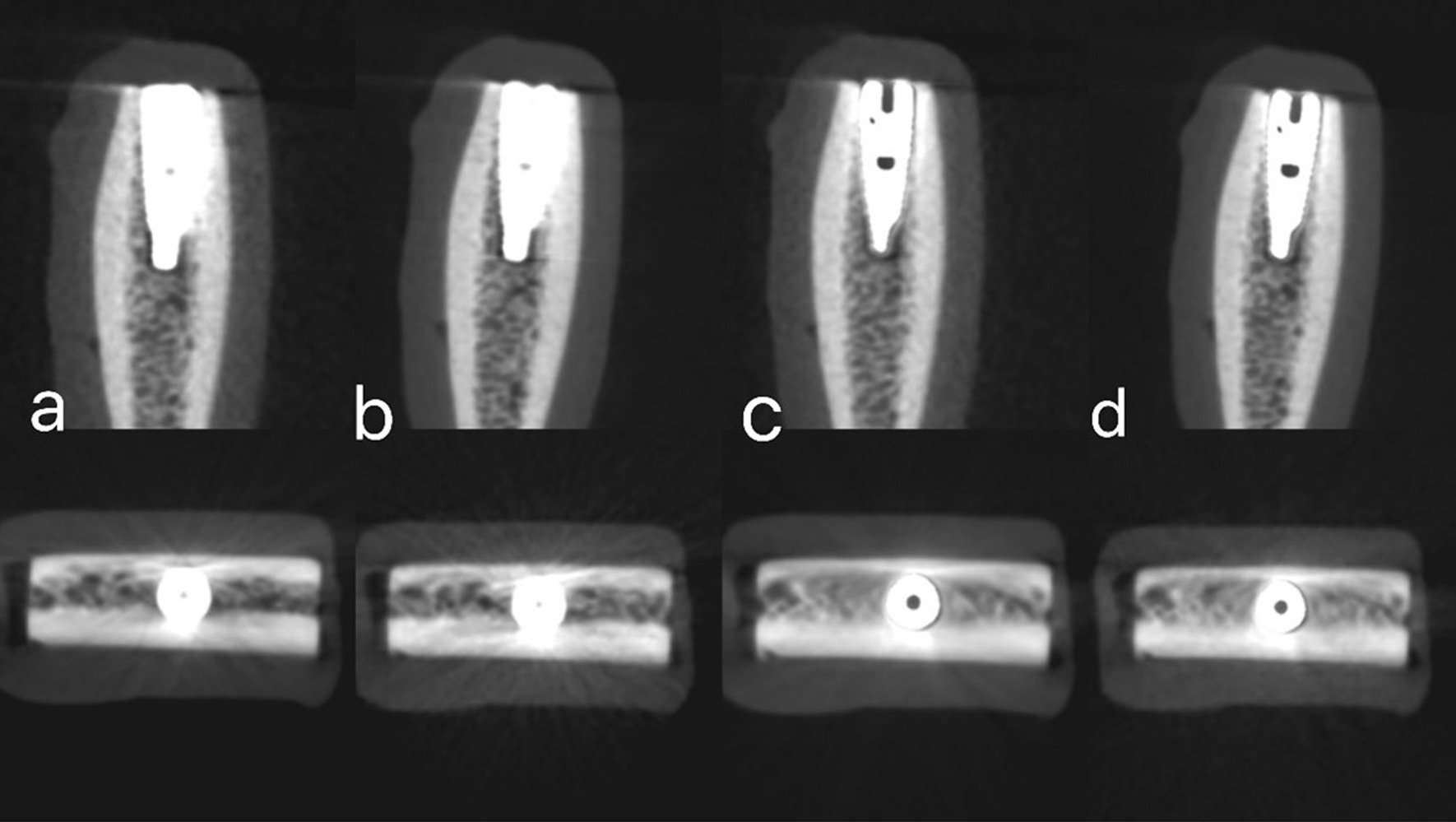
Sagittal and axial images with no peri-implant defect obtained with different four radiographic protocols. **a** High-dose CBCT without MAR. **b** Low-dose CBCT without MAR. **c** High-dose with MAR. **d** Low-dose CBCT with MAR

Cone beam computed tomography images were acquired using the Green Ct machine (Green Ct, Vatech, Hwaseong, Republic of Korea). Images were taken with two different exposure protocols: high-definition CBCT (HD-CBCT) and low-dose CBCT (LD-CBCT), once with the activation of the machine MAR tool and again without the MAR tool activation. So, each bone block was subjected to four radiographic examinations: HD with MAR, HD without MAR, LD with MAR, and LD without MAR. For HD-CBCT, the following parameters were adjusted: 5 × 5 cm 2 FOV, 90 kVp, 12 mA, 9 s scan time, and 0.08 mm voxel size with a 396.92 mGycm2 dose area product (DAP). For LD-CBCT, the parameters were as follows: 5 × 5 cm2 FOV, 90 kVp, 2 mA, 5.9 s scan time, and 0.08 mm voxel size with a 48.7 mGycm2 dose area product DAP. All bone blocks were scanned using each of the four radiographic protocols. The original CBCT images have been imported in the form of Digital Imaging and Communications in Medicine (DICOM) data for examination using Cybermed International's OnDemand3DTM version 1.0.10.4304 software (Seoul, Republic of Korea) (Figs. 3, 4, 5).

### Radiographic evaluation

A total of 120 datasets of CBCT images were examined by four examiners: three radiologists (one with more than 5 years’ experience and two with 3 years’ experience) and one periodontist. Prior to the initial evaluation, a calibration session was conducted using images from each radiographic protocol to standardize the radiological assessment of peri-implant defects. The image numbers were also shuffled to reduce the possibility of bias. Also, the analysis was performed in a new random sequence after 2 weeks to minimize learning bias and to obtain intra-examiner reliability.

During the evaluation sessions, the examiners were blinded and unaware of whether a defect was present or not, but they were allowed to scroll and view images of the entire volume in the three planes (axial, coronal, and sagittal), alter the brightness and contrast, and use the zoom tool. All images were viewed under the same conditions in a dimly lit room on a monitor with a 15.6" FHD 1920 × 1080 IPS display with an NVIDIA GeForce GTX 1650 graphic card. They assessed each image with MPR mode for the presence or absence of peri-implant bone defects using a five-point rank scale proposed by De-Azevedo-Vaz et al. [2]: “1-definitely absent, 2-probably absent, 3-uncertain, 4-probably present, 5-definitely present”.

### Statistical analysis

Data was analyzed using MedCalc Statistical Software version 19.0.5 (MedCalc Software bvba, Ostend, Belgium; https://www.medcalc.org; 2019) and significance was set at p value < 0.05. A receiver operating curve (ROC) was used to determine the diagnostic accuracy of the radiographic modalities, with multiple pairwise comparisons between the AUCs generated for the radiographic modalities using DeLong et al. [28] method. For all radiographic modalities used in the study, sensitivity (correctly identifying the presence of a defect), specificity (correctly identifying the absence of a defect), and test accuracy (percentage of correctness) were calculated using a five-point scale, where ranks 1, 2, and 3 representing absence of the defect, and ranks of 4 and 5 indicating its presence as it mentioned by Dave et al. [7]

## Results

The inter-observer reliability was calculated and, the intraclass correlation coefficient (ICC) ranged from 0.887 to 0.984, indicating very good to excellent agreement between observers in all the radiographic modalities. The intra-observer reliability was calculated and, the intra ICC ranged from 0.939 to 0.990 indicating excellent agreement across time (Table 1).

**Table 1:** Shows intra-observer and inter-observer reliability

			HD- CBCT without MAR	HD-CBCT with MAR	LD- CBCT without MAR	LD-CBCT with MAR
			Intraclass correlation coefficient			
Inter-observer reliability	Observer 1	Observer 2	0.971	0.897	0.975	0.935
		Observer 3	0.981	0.919	0.978	0.918
		Observer 4	0.984	0.902	0.980	0.950
	Observer 2	Observer 3	0.978	0.887	0.975	0.921
		Observer 4	0.984	0.918	0.967	0.920
	Observer 3	Observer 4	0.979	0.892	0.978	0.891
Intra-observer reliability	Observer 1		0.989	0.954	0.990	0.951
	Observer 2		0.977	0.952	0.984	0.951
	Observer 3		0.979	0.947	0.979	0.939
	Observer 4		0.985	0.972	0.989	0.949

The area under the ROC curve (AUC values) for detection of fenestration and dehiscence defects was calculated for both HD-CBCT and LD-CBCT with and without MAR tool (Tables 2, 3). AUC values were between 0.90 and 1.00 for the detection of fenestration defects and between 0.68 and 1.00 for dehiscence. The fenestration defect AUC values were higher than those for dehiscence defects when the MAR tool was applied. According to AUC values, application of the MAR tool with either HD-CBCT or LD-CBCT decreased the diagnostic accuracy for detection of both fenestration and dehiscence defects.

**Table 2:** Shows area under (ROC) curve (AUC), standard error (SE), confidence interval (CI) for fenestration group

	AUC	SE	95% CI of AUC
HD-CBCT without MAR	1.00	0.00	0.888, 1.00
LD-CBCT without MAR	1.00	0.00	0.888, 1.00
HD-CBCT with MAR	0.929	0.0391	0.776, 0.990
LD-CBCT with MAR	0.905	0.0439	0.744, 0.980
p-value	HD-CBCT without MAR versus LD-CBCT without MAR: 1.00 HD-CBCT without MAR versus HD-CBCT with MAR: 0.07 HD-CBCT without MAR versus LD-CBCT with MAR: 0.03* HD-CBCT with MAR versus LD-CBCT without MAR: 0.07 HD-CBCT with Mar versus LD-CBCT with MAR: 0.69 LD-CBCT without MAR versus LD-CBCT with MAR: 0.03*		

*Statistically significant at p-value ≤ 0.05

**Table 3:** Shows area under (ROC) curve (AUC), standard error (SE), confidence Interval (CI) for dehiscence group

	AUC	SE	95% CI of AUC
HD-CBCT without MAR	1.00	0.00	0.839, 1.00
LD-CBCT without MAR	1.00	0.00	0.839, 1.00
HD-CBCT with MAR	0.818	0.0761	0.591, 0.950
LD-CBCT with MAR	0.682	0.0761	0.445, 0.865
p-value	HD-CBCT without MAR versus LD-CBCT without MAR: 1.00 HD-CBCT without MAR versus HD-CBCT with MAR: 0.02* HD-CBCT without MAR versus LD-CBCT with MAR: < 0.001* HD-CBCT with MAR versus LD-CBCT without MAR: 0.02* HD-CBCT with Mar versus LD-CBCT with MAR: 0.21 LD-CBCT without MAR versus LD-CBCT with MAR: < 0.001*		

*Statistically significant at p-value ≤ 0.05

Sensitivity, specificity, and accuracy for all radiographic techniques were calculated independently for both fenestration and dehiscence defects (Table 4). For HD-CBCT and LD-CBCT without the application of MAR tool, all of these values were the same for fenestration and dehiscence. When the MAR tool was applied, the diagnostic accuracy for both scanning protocols was significantly decreased. However, the sensitivity, and accuracy values were higher for HD-CBCT than for LD-CBCT for both fenestration and dehiscence defect detection, while specificity values were the same in both cases. Also, the diagnostic values for fenestration defects were higher than those for dehiscence.

**Table 4:** Shows diagnostic values for fenestration and dehiscence

	Radiographic technique	Sensitivity (%)	Specificity (%)	Accuracy (%)
Fenestration	HD-CBCT without MAR	100	100	100
	LD-CBCT without MAR	100	100	100
	HD-CBCT with MAR	85.71	100	90.32
	LD-CBCT with MAR	80.95	100	87.10
Dehiscence	HD-CBCT without MAR	100	100	100
	LD-CBCT without MAR	100	100	100
	HD-CBCT with MAR	63.64	100	80.95
	LD-CBCT with MAR	40	100	70

## Discussion

Biomechanical complications and accumulation of bacterial biofilm are the main causative factors in the development of peri-implantitis and peri-implant bone defects. Fenestrations and dehiscences can affect the success of implant therapy and can subsequently result in progressive bone loss and implant failure. As a result, early detection of these defects is important for preserving the implants with the aid of radiographic examinations [7, 29]. According to several studies [3, 9, 10, 30], periapical and panoramic radiographs cannot be used for the evaluation of interproximal bone level around dental implants, and CBCT should be employed when peri-implant dehiscence or fenestration is suspected.

Bovine rib bone blocks were used in the current study because it was believed that the bone density and proportions between the cancellous and cortical bone in bovine ribs were similar to those in the mandible of humans. This was consistent with de‐Azevedo‐Vaz et al. [2], Saberi et al. [9], and Schwindling et al. [31], who used bovine rib models to evaluate peri-implant bone defects. For the selection of acquisition parameters, the smallest FOV of the machine was chosen as it was recommended by Pinheiro et al. [32] to decrease the artifacts from the surrounding tissues and optimize the radiation dose. Based on Vasconcelos et al. [33] findings, voxel size didn’t have an effect on beam hardening artifacts production next to titanium implants, so we chose a voxel size of 0.08 to enhance image quality and improve spatial resolution. For tube voltage, we choose 90 kVp for all radiographic protocols as it was suggested by Pauwels et al. [34], who reported that 90 kVp gives the optimum image quality and less noise than images obtained with 75 kVp.

For optimization of radiation dose, several previous studies [35–37] concluded that decreasing tube current in the presence of metallic objects may increase the magnitude of artifacts but has no significant effect on the diagnostic ability of CBCT images. Fontenele et al. [35] showed that decreasing the tube current had no effect on the detection accuracy of vertical root fractures in the endodontically treated teeth next to zirconium implants. Also, Sawicki et al. [36] found that the difference in tube current didn’t affect the assessment of peri-implant bone level. Based on these findings, we chose 12 mA (the highest mA offered by the CBCT machine used) for HD-CBCT and 2 mA (the least mA offered by the CBCT machine used) for LD-CBCT.

Low-dose CBCT protocols are associated with a significant decrease in radiation dose in comparison to high-dose protocols. For the current study, the dose area products of the protocols used were 48.7 mGycm2 for LD-CBCT and 396.92 mGycm2 for HD-CBCT. Despite the representation of the dose in the form of DAP, it still indicates that the use of the low-dose protocol significantly decreases the dose when compared with the high-definition protocol. Although the reduction in radiation dose of LD-CBCT protocols is significant, an insufficient amount of research is available for its application in implant assessment. A study conducted by Liljeholm et al. [38] showed that ultra-low dose protocols of CBCT can be used for pre-implant radiographic assessment. Another study held by Cardarelli et al. [39] stated that using a low-dose protocol with 180 degrees rotation angle has a significant effect on decreasing metal artifacts around the implants and can be used to assess peri-implant bone level. However, for detection of peri-implant dehiscence and fenestrations, de‐Azevedo‐Vaz et al. [5] recommended using the full scan protocol (360 degrees) for the detection of peri-implant dehiscence.

The results of our study showed that there was no statistically significant difference in fenestration and dehiscence detection between HD-CBCT and LD-CBCT when the MAR tool was not applied. Our study results support a previous study conducted by Schwindling et al. [31] to compare the accuracy of HD-CBCT and LD-CBCT in the detection and classification of peri-implant bone lesions, which stated that there was no significant difference in the diagnostic accuracy of both protocols. Also, in a study conducted by Schriber et al. [40] no difference was found between low-dose and high-dose protocols in the detection of buccal peri-implant dehiscence defects. Our results were in line with the results of Aktuna-Belgin et al. [41] who evaluated the efficiency of two different CBCT doses (low dose and ultra-low dose) in the detection of peri-implant fenestration and dehiscence defects, and they found that the diagnostic accuracy was not affected in the two protocols used.

The incorporation of MAR algorithms into CBCT units by manufacturers has resulted in a steady growth in the utilization of these approaches. Different previous studies evaluated the effects of the MAR algorithm on the artifacts of CBCT images, and they stated that it can reduce the standard deviations of grey value and increase image quality by increasing contrast to noise ratio (CNR) [42–45]. The application of these strategies has been examined, but the findings have been uneven. Fontenele et al. [46] found that the MAR tool has a negative effect on the diagnostic accuracy of vertical root fracture in the presence of intracanal filling. Also, Kamburoglu et al. [47] evaluated the effects of four MAR protocols (off, low, medium, and high) for the assessment of periodontal and peri-implant defects, and they found no difference in diagnostic accuracy with any MAR protocol. However, Bagis et al. [48] recommended the use of the MAR tool for the detection of peri-implant fenestration defects.

According to the results of the current study, using the MAR tool decreased AUC, sensitivity, and accuracy values when applied to both HD-CBCT and LD-CBCT. Only specificity values were the same whether MAR tool was applied or not. It means that the detection of true defect blocks was significantly decreased by the application of MAR tool, while the no defect intact blocks could be detected correctly with and without the application of MAR tool with no difference. This was consistent with the findings of both De-Azevedo-Vaz et al. [2] and Sheikhi et al. [28]. De-Azevedo-Vaz et al. [2] stated that the MAR algorithm didn’t improve the diagnostic accuracy of fenestration and dehiscences. Sheikhi et al. [23] found that sensitivity and accuracy values for fenestration and dehiscence defects were higher when the MAR algorithm was absent, but specificity was equal when MAR was present and absent, which was almost similar to our findings.

In the current study, when MAR tool was applied, the AUC values and the diagnostic values revealed that peri-implant fenestrations were more correctly diagnosed than peri-implant dehiscences. Similar findings have been reported by de-Azevedo-Vaz et al. [2, 5], Sheikhi et al. [23], and Salemi et al. [26], and they explained these findings by claiming that as dehiscence has just an inferior border, it is more difficult to identify than fenestration, which has both superior and inferior borders.

MAR algorithms employ several methods to minimize metal artifacts, such as iterative reconstruction methods, projection-correction methods, and reconstruction-correction methods. The majority of these methods consider metal artifacts as missing data [49, 50]. The technique of the MAR algorithm of the CBCT machine used in the study was not explained by the manufacturer. Unfortunately, the disadvantages of these techniques include the elimination of all attenuation data from high-density objects. When using the MAR algorithm, there is always lost data that cannot be reconstructed, which may cause modification in the image and can affect the diagnostic accuracy [43, 51]. Also, according to Fontenele and Mancini et al. [42, 52] the impact of the MAR algorithm becomes more prominent when the image artifacts are increased. Hence, it explains the decrease in the diagnostic ability of LD-CBCT compared with HD-CBCT when the MAR tool was applied to both in the current study, as we changed the tube current from 12 mA in HD-CBCT to 2 mA in LD-CBCT. Thus, the production of artifacts from the implants was increased [52, 53].

The in vitro design is one of the limitations of our study. In clinical situations, patient movement artifacts may affect the quality of the final image and affect the diagnostic accuracy, but this is the only ethically appropriate protocol to assess the recommended parameters without subjecting patients to unneeded CBCT scans[19]. Also, the presence of metal restorations, teeth, and surrounding tissues in the clinical situations may make the detection of these defects more difficult. Another limitation is that peri-implant dehiscence and fenestration defects were prepared using a diamond bur with a definite border and differed from the naturally occurring defects, which have tapered borders and are more difficult to detect [54].

## Conclusion

Within the limitations of the study and the CBCT acquisition parameters used, LD-CBCT can be used without any decrease in diagnostic accuracy for the detection of peri-implant fenestration and dehiscence defects. The use of the MAR algorithm reduces the diagnostic ability of both CBCT scanning protocols for the detection of both peri-implant defects, especially for the dehiscence defect.

## Data Availability

All data included in this study are available from the corresponding author upon request.

## Abbreviations

CBCT: Cone beam computed tomography
LD-CBCT: Low dose cone beam computed tomography
HD-CBCT: High-definition cone beam computed tomography
MAR: Metal artifact reduction tool
ROC curve: Receiver operating characteristic curve
AUC: Area under curve
DAP: Dose area product
